# 原发性血小板增多症患者生存预后积分系统预后价值的比较研究：IPSET、MIPSS-ET、AAA和AAA+

**DOI:** 10.3760/cma.j.cn121090-20260131-00072

**Published:** 2026-06

**Authors:** 颂扬 赵, 泽锋 徐, 铁军 秦, 冰 李, 士强 曲, 琦 孙, 玉娇 贾, 承文 李, 丽娟 潘, 清妍 高, 蒙 焦, 泽飞 包, 志坚 肖

**Affiliations:** 1 北京协和医学院，中国医学科学院血液病医院（中国医学科学院血液学研究所），血液与健康全国重点实验室，国家血液系统疾病临床医学研究中心，细胞生态海河实验室，天津 300020 State Key Laboratory of Experimental Hematology, National Clinical Research Center for Blood Diseases, Haihe Laboratory of Cell Ecosystem, Institute of Hematology & Blood Diseases Hospital, Chinese Academy of Medical Sciences & Peking Union Medical College, Tianjin 300020, China; 2 天津医学健康研究院，天津 301600 Tianjin Institutes of Health Science, Tianjin 301600, China

**Keywords:** 原发性血小板增多症, 生存, 预后积分系统, Essential thrombocythemia, Survival, Prognostic scoring systems

## Abstract

**目的:**

验证并比较原发性血小板增多症国际预后积分系统（IPSET）、包含基因突变的原发性血小板增多症预后积分系统（MIPSS-ET）、AAA和AAA+在中国原发性血小板增多症（ET）患者中的适用性。

**方法:**

本研究为临床预测模型的外部验证比较研究，对2015年1月至2022年12月就诊于中国医学科学院血液病医院的423例ET患者进行回顾性分析，分别采用IPSET、MIPSS-ET、AAA和AAA+生存预后积分系统进行风险分层，并采用一致性指数（C-index）、Brier得分、净重新分类指数（NRI）和综合判别改善指数（IDI）综合评估并比较各模型对总生存（OS）的预测区分度、校准度与危险度再分层效能。

**结果:**

纳入患者诊断时中位年龄50（*IQR*：34，62）岁，女性占60.8％（257例）。22.0％（93例）有诊断前血栓史。JAK2V617F、CALR及MPL驱动基因突变检出率分别为56.7％（240例）、17.0％（72例）和5.4％（23例），21.3％（90例）患者未检出驱动基因突变。中位随访63.1（95％*CI*：60.9～68.5）个月，随访期间共16例（3.8％）患者死亡，5年OS率为（97.4±0.8）％。IPSET、MIPSS-ET、AAA及AAA+四种积分系统均能有效区分不同风险组的OS（均*P*<0.001）；各系统预测OS的C-index分别为0.756、0.775、0.762和0.823，5年Brier得分分别为0.023 5、0.022 7、0.023 2和0.022 6。在危险度再分层方面，AAA+相较于IPSET、MIPSS-ET和AAA预后积分系统的NRI分别为0.486、0.352和0.268，能将部分被其他系统判定为低/中危的患者准确重新归入更高风险组，且这部分危险度上调患者的实际OS显著劣于未上调组。

**结论:**

IPSET及MIPSS-ET、AAA和AAA+生存预后积分系统均可有效评估中国ET患者的生存风险；其中，AAA+系统在OS预测上展现出最优的区分度、校准度，以及更准确的危险度再分层效能，更适用于中国ET人群的个体化生存预后评估。

## Abstract (en)

**Objective:**

To validate and compare the applicability of the international prognostic score for ET（IPSET）, the mutation-enhanced international prognostic systems for ET（MIPSS-ET）, triple A（AAA）, and triple A plus（AAA+）survival prognostic scoring systems in Chinese patients with essential thrombocythemia（ET）.

**Methods:**

In this external validation and comparative study of clinical prediction models, 423 patients with ET who visited the Institute of Hematology & Blood Diseases Hospital, Chinese Academy of Medical Sciences & Peking Union Medical College between January 2015 and December 2022 were retrospectively analyzed. Risk stratification was performed using the IPSET, MIPSS-ET, AAA, and AAA+ survival prognostic scoring systems. Harrell's concordance index（C-index）, Brier score, net reclassification improvement（NRI）, and integrated discrimination improvement（IDI）were used to comprehensively evaluate and compare the predictive discrimination, calibration, and risk restratification efficacy of each model for overall survival（OS）.

**Results:**

The median age at diagnosis of the enrolled patients was 50 years［interquartile range（*IQR*）: 34–62］, and 60.8％（257 cases）were female. Overall, 22.0％（93 cases）of the patients had a history of thrombosis prior to diagnosis. The mutation frequencies of the driver genes JAK2V617F, CALR, and MPL were 56.7％（240 cases）, 17.0％（72 cases）, and 5.4％（23 cases）, respectively, while 21.3％（90 cases）of the patients had no driver gene mutations. After a median follow-up of 63.1（95％ *CI*: 60.9–68.5）months, 16 patients（3.8％）died during the follow-up period, with an estimated 5-year OS rate of（97.4±0.8）％. IPSET, MIPSS-ET, AAA, and AAA+ scoring systems could effectively discriminate the OS of patients in different risk groups（all *P*<0.001）; the C-index for OS prediction was 0.756 for IPSET, 0.775 for MIPSS-ET, 0.762 for AAA, and 0.823 for AAA+, and the 5-year Brier scores were 0.023 5, 0.022 7, 0.023 2, and 0.022 6, respectively. Regarding risk restratification, the NRI of the AAA+ scoring system compared with the IPSET, MIPSS-ET, and AAA systems were 0.486, 0.352, and 0.268, respectively. The AAA+ system could accurately reclassify some patients who were categorized as low- or intermediate-risk by other systems into higher-risk groups, and the actual OS of these up-stratified patients was significantly inferior to that of the non-up-stratified group.

**Conclusion:**

The IPSET, MIPSS-ET, AAA, and AAA+ survival prognostic scoring systems are all effective for assessing survival risk in Chinese patients with ET. Among them, the AAA+ system exhibited optimal discrimination, calibration, and more accurate risk restratification efficacy in OS prediction, making it more suitable for individualized survival prognostic assessment in the Chinese ET population.

原发性血小板增多症（Essential thrombocythemia, ET）是经典费城染色体阴性的骨髓增殖性肿瘤（Myeloproliferative neoplasms, MPN）中最常见的亚型[1]–[2]。尽管ET患者整体预期寿命接近同龄正常人群，但其临床异质性显著，部分患者可能经历骨髓纤维化或急性白血病转化，从而导致总生存（OS）期显著缩短。因此，准确评估ET患者的OS风险，对于制定个体化随访与治疗策略至关重要。

针对ET患者OS的预后积分系统不断演进。早期的国际预后积分系统（International prognostic scoring system for ET, IPSET）主要依赖年龄、白细胞计数及血栓病史等临床表型特征[3]。随着分子诊断的普及，结合了预后不良基因突变状态的ET预后积分系统（Mutation-enhanced international prognostic system for ET, MIPSS-ET）被提出，进一步提升了风险分层的精准度[4]。近年，中性粒细胞绝对计数、淋巴细胞绝对计数以及患者自身相关因素，如性别、心血管危险因素对患者预后的影响逐渐被重视，Tefferi等[5]–[6]又相继提出了AAA和AAA+预后积分系统。

上述生存预后模型均基于西方患者队列开发。目前，这些新型预后系统（特别是AAA和AAA+）在中国人群中的适用性尚无大样本数据的独立验证，各模型间的预测效能亦缺乏直接的头对头比较。

基于此，本研究依据《个体预后或诊断的多变量预测模型透明报告》（Transparent reporting of a multivariable prediction model for individual prognosis or diagnosis, TRIPOD）规范，回顾性收集本中心423例ET患者的临床与分子生物学数据，旨在对AAA、AAA+、IPSET及MIPSS-ET四个生存预后积分系统进行外部验证，综合评价并比较各模型的区分度与校准度，从而评估适合我国ET患者的预后积分系统。

## 病例与方法

1. 病例：本研究纳入2015年1月至2022年12月就诊于中国医学科学院血液病医院MDS和MPN诊疗中心的ET患者，纳入标准：①符合WHO第五版诊断分型标准[7]；②新诊断或诊断1年内且未接受降细胞药物治疗；③有血液肿瘤相关基因二代测序资料。共423例患者纳入本研究。本研究经中国医学科学院血液病医院伦理委员会批准（批件号：KT2020035-EC-2），所有患者均知情同意，本研究符合《赫尔辛基宣言》的要求。

2. 染色体核型：骨髓细胞培养24 h后，收集细胞、常规制片，G显带，染色体核型描述按照《人类细胞遗传学国际命名体制（ISCN2013）》[8]。

3. 血液肿瘤相关基因二代测序：留取患者的外周血或骨髓样本，分离出单个核细胞并提取DNA。使用PCR引物扩增目的基因组（包含15～267个血液系统肿瘤相关基因），对目的区域DNA富集，采用Illumina测序平台测序。测序后的原始数据利用CCDS、人类基因组数据库（HG38）、dbSNP（v138）、1000genomes、COSMIC、PolyPhen-2等数据库进行生物信息学分析，筛选出致病性/可能致病性基因突变位点。为减少缺失数据对统计分析的影响，仅对在总体队列中基因检测缺失比例<10％的基因进行分析。

4. 生存相关预后积分系统评分：

IPSET[3]：年龄≥60岁（2分）、WBC≥11×10^9^/L（1分）、血栓病史（1分）。依累计积分分组：低危组（0分），中危组（1～2分），高危组（≥3分）。

MIPSS-ET[4]：年龄>60岁（4分）、男性（1分）、WBC≥11×10^9^/L（1分）、预后不良基因突变（SRSF2、SF3B1、U2AF1和TP53）（2分），分为低危组（0～1分）、中危组（2～5分）和高危组（≥6分）。

AAA[5]：年龄（>70岁赋值4分，50～70岁赋值2分）、外周血淋巴细胞绝对计数<1.7×10^9^/L（1分）、外周血中性粒细胞绝对计数≥8×10^9^/L（1分），分为低危组（0～1分）、中危-1组（2～3分）、中危-2组（4分）和高危组（5～6分）。

AAA+[6]：年龄（>70岁赋值8分，50～70岁赋值2分）、外周血中性粒细胞绝对计数≥8×10^9^/L（1分）、外周血淋巴细胞绝对计数<1.7×10^9^/L（1分）、外周血单核细胞绝对计数≥0.5×10^9^/L（1分）、男性（1分）、高血压病史（1分）、动脉血栓病史（1分），分为极低危组（0～1分）、低危组（2～4分）、中危组（5分）和高危组（6～14分）。

5. 治疗：389例（92.0％）患者随访到治疗方案，40例（10.3％）观察和等待，78例（20.1％）仅接受抗血小板药物治疗，152例（39.1％）和121例（31.1％）接受了羟基脲和干扰素α治疗。

6. 随访：末次随访截止时间为2025年11月20日，中位随访63.1（95％*CI*：60.9～68.5）个月。随访资料来自门急诊病历、住院病历或电话随访记录。对随访期间死亡病例，根据病历或与患者家属电话联系确认。OS期定义为疾病诊断至死亡或末次随访时间。

7. 统计学处理：非正态分布的连续性变量用中位数（*IQR*）描述，分类变量采用例（％）描述。缺失数据直接剔除该病例。利用Mann-Whitney *U*检验比较非正态分布的连续性变量差异，采用卡方检验或Fisher确切概率法比较分类变量差异。Kaplan-Meier法绘制生存曲线，利用Log-rank检验进行分析。区分度采用Harrell's一致性指数（C-index）进行评估，其95％*CI*及各模型之间C-index差异的统计学意义通过Bootstrap法（重抽样1 000次）进行成对验证与检验。校准度评估通过计算Brier分数进行评估比较。此外，为量化不同预后积分系统在临床实际人群中的危险度再分层效能，本研究计算了分类净重新分类指数（Categorical Net Reclassification Improvement, NRI）与综合判别改善指数（Integrated Discrimination Improvement, IDI）。NRI用于评估新模型将患者正确归入更高或更低风险等级的净比例；IDI用于评估新模型相较于旧模型在连续生存预测概率层面区分事件组与非事件组能力的提升情况。对于重新分层的特定亚组患者（如旧模型判定为低中危但新模型判定为高危的患者），采用Kaplan-Meier法绘制生存曲线，并通过Log-rank检验比较其总生存差异。双侧*P*<0.05为差异有统计学意义。统计分析利用SPSS 27.0和R 4.3.2软件完成，应用Graphpad Prism 10.0及R 4.3.2绘图。

## 结果

一、ET患者的基本特征与转归

1. 临床和实验室特征：423例患者纳入本研究，其临床和实验室特征见表1。总体而言，本队列以中年起病为主，且女性多于男性。在预后模型相关的核心危险因素中，近两成患者初诊时存在白细胞或中性粒细胞显著增多，超过三分之一的患者伴有心血管危险因素或淋巴细胞绝对计数减低。此外，22.0％（93/423）的患者在诊断前已发生血栓事件（以动脉血栓为主）。本队列绝大多数患者的临床指标完整，虽然LDH（27.6％，117/423）与染色体核型（13.5％，57/423）存在一定比例缺失，但均非模型相关指标；基本满足AAA、AAA+、IPSET及MIPSS-ET预后积分系统的分层计算需求。

**表1:** 原发性血小板增多症患者的临床和实验室特征

变量	值
诊断时年龄［岁，*M*（*Q*_1_，*Q*_3_）］	50（34，62）
女性［例（％）］	257（60.8）
WBC［×10^9^/L，*M*（*Q*_1_，*Q*_3_）］	8.5（7.0，10.3）
WBC≥11×10^9^/L［例（％）］	79（18.7）
ANC［×10^9^/L，*M*（*Q*_1_，*Q*_3_）］（422例）	5.8（4.5，7.4）
ANC≥8×10^9^/L［例（％）］（422例）	76（18.0）
ALC［×10^9^/L，*M*（*Q*_1_，*Q*_3_）］（421例）	1.9（1.5，2.4）
ALC<1.7×10^9^/L［例（％）］（421例）	145（34.4）
单核细胞绝对计数［×10^9^/L，*M*（*Q*_1_，*Q*_3_）］（422例）	0.4（0.3，0.5）
HGB［g/L，*M*（*Q*_1_，*Q*_3_）］	141（130，149）
PLT［×10^9^/L，*M*（*Q*_1_，*Q*_3_）］	756（617，978）
PLT≥1 000×10^9^/L［例（％）］	97（22.9）
LDH［U/L，*M*（*Q*_1_，*Q*_3_）］（306例）	217（186，256）
体质性症状［例（％）］	14（3.3）
心血管危险因素［例（％）］（402例）	156（38.8）
脾左肋缘下可触及［例（％）］（421例）	26（6.2）
循环中原始细胞比例≥1％［例（％）］	1（0.2）
诊断前血栓史［例（％）］^a^	93（22.0）
诊断前动脉血栓病史	88（20.8）
诊断前静脉血栓病史	6（1.4）
骨髓纤维化分级［例（％）］	
0级	231（54.6）
1级	192（45.4）
染色体核型［例（％）］（366例）	
异常核型	8（2.2）
复杂核型	0（0）
诊断后血栓［例（％）］	37（8.7）
诊断后动脉血栓病史	30（7.1）
诊断后静脉血栓病史	7（1.7）

**注** ANC：中性粒细胞绝对计数；ALC：淋巴细胞绝对计数；LDH：乳酸脱氢酶；^a^1例患者同时具有诊断前动脉和静脉血栓病史

2. 细胞遗传学和分子生物学特征：366例患者有可供分析的染色体核型，其中8例（2.2％）有克隆性染色体核型异常，未检出复杂染色体核型。240例（56.7％）患者检出JAK2V617F基因突变；72例（17.0％）检出CALR基因突变，type1/1-like突变42例（9.9％），type2/2-like突变29例（6.9％）；23例（5.4％）检出MPL基因突变；其中1例（0.2％）患者同时检出JAK2V617F和CALR基因突变，1例（0.2％）患者同时检出JAK2V617F和MPL基因突变，90例（21.3％）患者未检出驱动基因突变。JAK2V617F、CALR、MPL中位等位基因频率（Variant allelic frequency，VAF）分别为18.1％（*IQR*：11.4％，26.2％），17.5％（*IQR*：10.0％，39.5％）和25.0％（*IQR*：12.5％，34.6％）。常见非驱动基因突变依次为：TET2（8.5％，36/423），DNMT3A（8.3％，35/423），ASXL1（2.9％，12/420），NOTCH2（2.6％，10/389），KMT2D（2.3％，9/389），FAT1（2.1％，8/389），SF3B1（1.7％，7/420），RELN（1.5％，6/389），SRSF2（1.2％，5/420），TP53（1.2％，5/423）。19例（4.5％）患者检出至少1个ET预后不良基因突变［SRSF2（5/420，1.2％）、SF3B1（7/420，1.7％）、U2AF1（3/420，0.7％）和TP53（5/423，1.2％）］。

3. 转归：全部423例有随访资料，中位随访63.1（95％*CI*：60.9～68.5）个月，随访过程中2例（0.5％）进展为ET后骨髓纤维化，3例（0.7％）进展为急性白血病；16例（3.8％）死亡。其中9例明确了具体死亡原因（血栓3例，出血2例，感染1例，进展白血病、骨髓纤维化和其他肿瘤各1例）。总体患者5年OS率为（97.4±0.8）％。

二、不同生存预后积分系统危险度分组分布（图1）

1. IPSET：423例患者根据IPSET生存预后积分系统，低危组209例（49.4％）、中危组146例（34.5％）、高危组68例（16.1％），三组的5年OS率分别为（99.4±0.6）％、（96.5±1.5）％和（92.8±3.5）％（*χ*^2^＝23.37，*P*<0.001）（图2A）。

**图1:**
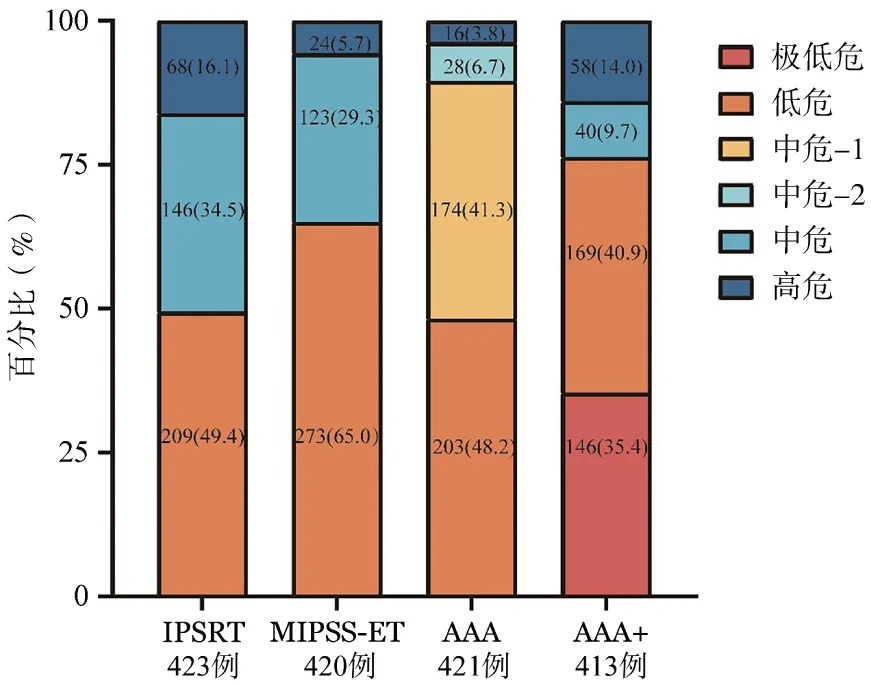
原发性血小板增多症患者不同生存预后积分系统危险度分组分布 **注** IPSET：原发性血小板增多症国际预后积分系统；MIPSS-ET：包含基因突变的原发性血小板增多症预后积分系统

**图2:**
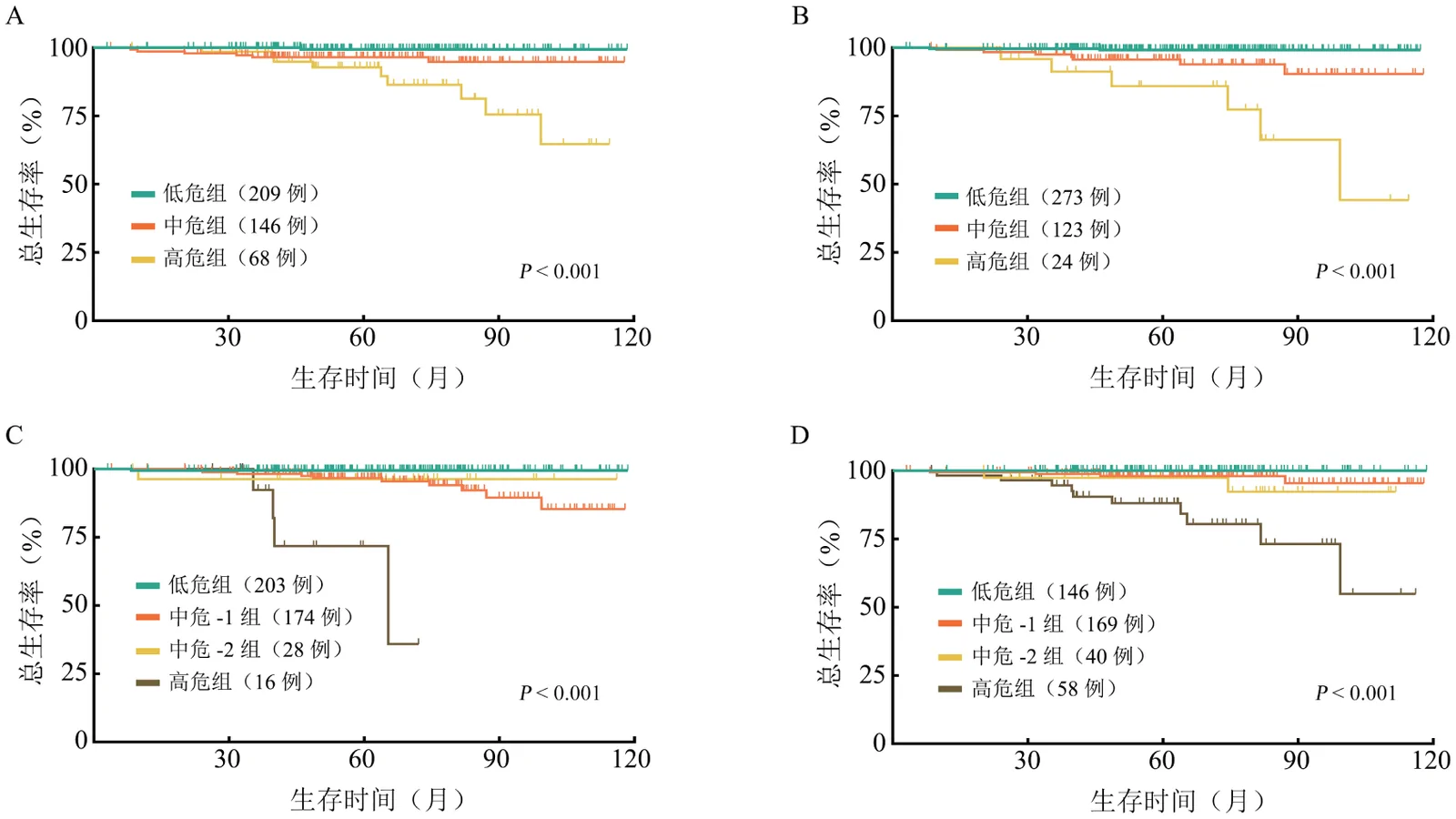
原发性血小板增多症患者不同预后积分系统总生存率比较 **A** IPSET；**B** MIPSS-ET；**C** AAA；**D** AAA+ **注** IPSET：原发性血小板增多症国际预后积分系统；MIPSS-ET：包含基因突变的原发性血小板增多症预后积分系统

2. MIPSS-ET：基于MIPSS-ET生存预后积分系统分组的420例患者中，低危组273例（65.0％）、中危组123例（29.3％）、高危组24例（5.7％），三组的5年OS率分别为（99.2±0.6）％、（95.6±1.9）％和（85.9±7.6）％（*χ*^2^＝38.29，*P*<0.001）（图2B）。

3. AAA：421例患者可按照AAA生存预后积分系统进行风险分层，低危组占48.2％（203/421）、中危-1组占41.3％（174/421）、中危-2组占6.7％（28/421）、高危组占3.8％（16/421），四组对应的5年OS率分别为（99.5±0.5）％、（96.7±1.5）％、（96.3±3.6）％和（71.8±14.0）％（*χ*^2^＝48.50，*P*<0.001）（图2C）。

4. AAA+：413例患者根据AAA+生存预后积分系统进行风险分层，极低危组占35.4％（146/413）、低危组占40.9％（169/413）、中危组占9.7％（40/413）、高危组占14.0％（58/413），四组对应的5年OS率分别为100％、（98.0±1.1）％、（97.4±2.5）％和（88.1±4.6）％（*χ*^2^＝39.64，*P*<0.001）（图2D）。

三、不同预后积分系统预后效力比较

IPSET、MIPSS-ET、AAA、AAA+预后积分系统OS的预测区分度及校准度评估显示，C-index分别为0.756（95％ *CI*：0.661～0.852）、0.775（95％ *CI*：0.654～0.896）、0.762（95％ *CI*：0.641～0.883）及0.823（95％ *CI*：0.736～0.911）；5年Brier得分分别为0.023 5、0.022 7、0.023 2及0.022 6；AAA+预后积分系统展现出最优的整体预测趋势。

比较AAA+预后积分系统与其他预后积分系统的危险度分层结果（表2）发现，在IPSET低危和中危组患者中，分别有5例（2.4％）和24例（16.4％）患者经AAA+重新分层后被归入更高风险组，即共29例患者发生危险度分组上调，并且上调组患者较未上调组患者预后差（图3A）。同样，在MIPSS-ET和AAA预后积分系统定义的低中危患者中，分别有56例和42例患者经AAA+重新分层后被归入更高风险组，上调组患者的OS均显著劣于未上调组患者（图3B、3C）。

**表2:** IPSET、MIPSS-ET、AAA和AAA+预后积分系统各组患者分布比较［例（％）］

预后积分系统危险度分组		AAA+				
		极低危	低危	中危	高危	缺失
IPSET	低危	130（62.2）	68（32.5）	5（2.4）	0（0）	6（2.9）
	中危	16（11.0）	83（56.8）	20（13.7）	24（16.4）	3（2.1）
	高危	0（0）	18（26.5）	15（22.1）	34（50.0）	1（1.5）
MIPSS-ET	低危	139（50.9）	107（39.2）	14（5.1）	7（2.6）	6（2.2）
	中危	6（4.9）	57（46.3）	21（17.1）	35（28.5）	4（3.3）
	高危	0（0）	4（16.7）	5（20.8）	15（62.5）	0（0）
AAA	低危	146（71.9）	52（25.6）	0（0）	0（0）	5（2.5）
	中危-1	0（0）	114（65.5）	35（20.1）	22（12.6）	3（1.7）
	中危-2	0（0）	3（10.7）	5（17.9）	20（71.4）	0（0）
	高危	0（0）	0（0）	0（0）	16（100）	0（0）

**注** IPSET：原发性血小板增多症国际预后积分系统；MIPSS-ET：包含基因突变的原发性血小板增多症预后积分系统

**图3:**
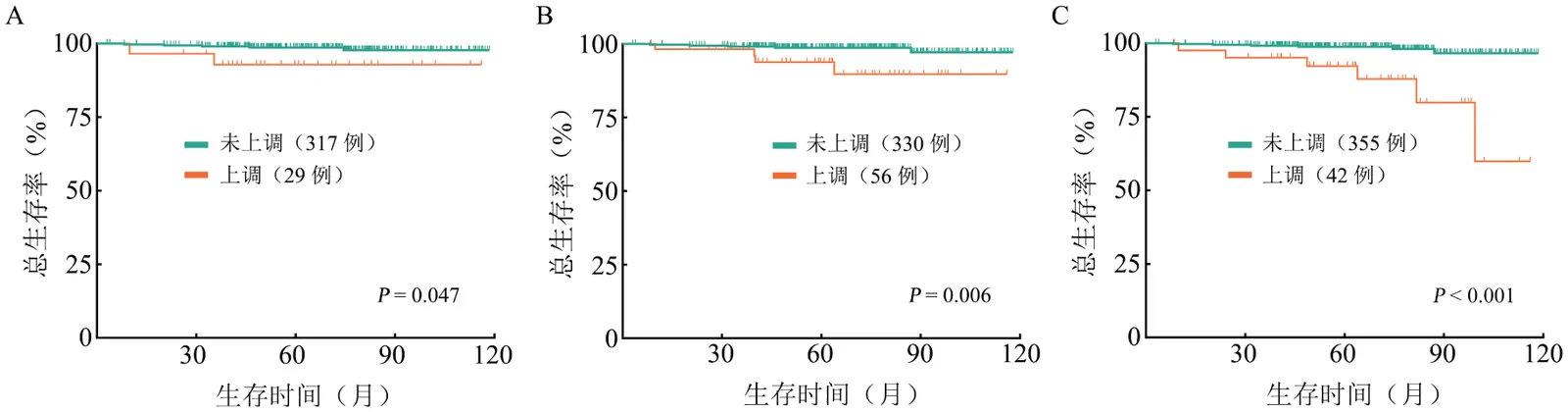
IPSET（A）、MIPSS-ET（B）、AAA（C）预后积分系统低危和中危患者经AAA+重新分层后预后危险度分组上调组与未上调组的总生存比较 **注** IPSET：原发性血小板增多症国际预后积分系统；MIPSS-ET：包含基因突变的原发性血小板增多症预后积分系统

以AAA+预后积分系统作为新模型，其相较于IPSET、MIPSS-ET和AAA预后积分系统的NRI分别为0.486、0.352和0.268（均>0）。这表明AAA+预后积分系统在患者风险等级的重新划分上具有显著的正向净获益，能够更准确地将高死亡风险的患者归入较高危组。在5年死亡风险预测概率的IDI方面，AAA+相较于IPSET和AAA亦表现出正向改善（IDI分别为0.022和0.007），而在与MIPSS-ET的对比中无明显劣势（IDI＝−0.011）。综合C-index与重分类指标，AAA+预后积分系统在本队列中展现出了更优越的临床预后判别与风险分层效力。

## 讨论

长期以来，患者确诊后的临床管理主要聚焦于血栓风险评估，而对其OS预后的关注直到近10年才逐渐引起重视。近年来，国际上先后提出了IPSET[3]、MIPSS-ET[4]、AAA[5]和AAA+[6]等多个ET生存预后积分系统，然而，这些模型均主要基于西方人群队列构建，考虑到不同种族间可能存在的遗传背景与临床表型差异，其在亚洲人群中的有效性尚待考证。本研究基于单中心423例ET患者的回顾性队列，在国内首次系统评估并比较了上述生存预后积分系统在中国ET患者中的适用性与预测效能。

IPSET基于年龄、白细胞计数和血栓病史构建。然而，随着对ET分子生物学特征认识的不断深入，研究显示SRSF2、SF3B1、U2AF1和TP53等非驱动基因与ET患者的不良结局密切相关，被定义为ET预后不良基因突变，并与男性这一性别因素共同被纳入MIPSS-ET预后积分系统中。新的研究提示，除分子生物学层面因素外，中性粒细胞绝对计数升高、淋巴细胞绝对计数减少不仅在骨髓增生异常肿瘤等髓系肿瘤提示预后不良，在ET患者中也与生存不良相关[5]–[6],[9]–[10]，这一发现促使ET预后积分系统中预后不良因素从白细胞计数向白细胞分类计数转变。因此，不同于IPSET和MIPSS-ET仅将白细胞计数升高作为不良预后因素，AAA和AAA+进一步细化了中性粒细胞绝对计数、淋巴细胞绝对计数及单核细胞绝对计数在预后评估中的价值。此外，AAA及AAA+对年龄的分层更加精细。IPSET将血栓史作为预后不良的危险因素，但未区分动脉血栓病史与静脉血栓病史的差异，且血栓病史的预后意义在不同人群中存在争议。相比之下，AAA+进一步细化，仅将动脉血栓病史纳入生存预后积分系统，体现了AAA+预后积分系统危险因素进一步明确，可能更好地预测ET患者的临床病程。

本研究结果显示，在中国ET患者中，AAA+在OS预测方面展现出最优的综合效能。其C-index为0.823，高于AAA、IPSET及MIPSS-ET，且Brier得分最低，提示其具备良好的区分度与预测校准度。这一发现与Tefferi等[6]在西方ET人群中得出的AAA+模型具有优越性的结论高度一致，进一步证实了该评分系统在跨种族人群中的广泛适用性。更重要的是，结合重分类分析（NRI>0），我们证实AAA+能够更精准地识别出被其他传统系统（如IPSET和MIPSS-ET）误判为低、中危的高危患者（图3）。这种预后风险的有效上调，有助于临床医师及早发现潜在的生存不良群体，从而为其制定更为积极、个性化的随访或干预策略。

值得注意的是，本研究中4个预后积分系统均识别出高危组患者OS显著差于其他预后危险度分组患者。目前临床实践中，ET患者的治疗决策主要基于患者的血栓风险分组，对生存预后的评估关注不足[1],[11]。本研究结果提示，在ET患者的临床管理中，将生存预后积分系统危险度分层与血栓风险评估相结合，有助于更全面地识别预后不良的高危人群，为治疗决策的优化提供依据。尽管目前尚缺乏证据表明现有药物治疗能够显著改善ET患者的长期生存结局[1]，但近年来，干扰素α治疗（尤其是聚乙二醇干扰素α和罗培干扰素α）在ET治疗中逐渐受到关注，其不仅能够有效控制血细胞计数，还可降低JAK2V617F等驱动基因的突变负荷，干扰素α治疗带来的分子学改善提示其或能让高危ET患者生存获益，但仍需大样本、长期随访的临床研究验证[1],[12]–[13]。既往关于芦可替尼治疗ET的临床研究结果提示[14]，与最佳可行治疗方案相比，芦可替尼在改善症状和缩小脾脏方面具有一定优势，但在完全缓解率、血栓和出血发生率以及疾病进展率方面未显示出显著获益。因此，目前尚无确切证据表明高危ET患者能够从芦可替尼治疗中获益。靶向CALR基因突变的单克隆抗体[15]以及赖氨酸特异性去甲基酶1（LSD1）抑制剂[16]等新型治疗策略已进入临床试验阶段，为高危ET患者提供了更多治疗选择。因此，在实际的临床决策过程中，应将血栓风险评估与生存预后积分系统相结合，早期识别生存预后不良的高危组患者，并制定更加合理的随访策略和治疗方案，以期改善该患者群体的预后。

本研究亦存在一定局限性。首先，本研究为单中心回顾性分析，难以完全避免选择偏倚。其次，尽管本研究中位随访时间已达63.1个月，但ET总体病程相对惰性，目前的随访跨度尚不足以对远期生存终点进行充分评估。相关结论仍需多中心、前瞻性及长期随访研究加以验证。

综上所述，AAA、AAA+、IPSET和MIPSS-ET生存预后积分系统均能有效评估中国ET患者的生存风险，其中AAA+在OS预测方面表现出更优的预测区分度与临床适用性，可能更适用于中国ET人群的预后评估，为个体化治疗决策及随访策略的制定提供重要参考。
